# Challenges with drug management of children and adolescents living with human immunodeficiency virus on antiretroviral therapy: A narrative review

**DOI:** 10.4102/phcfm.v18i1.5121

**Published:** 2026-06-29

**Authors:** Phumelele P. Mthethwa, Panjasaram Naidoo, Myra Taylor

**Affiliations:** 1 Discipline of Pharmaceutical Sciences, College of Health Sciences, University of KwaZulu-Natal, Durban, South Africa; 2 Discipline of Public Health Medicine, College of Health Sciences, University of KwaZulu-Natal, Durban, South Africa

**Keywords:** HIV/AIDS, challenges, drug, management, paediatric, patients, antiretroviral, therapy

## Abstract

**Background:**

Tremendous efforts and resources have been outlaid for human immunodeficiency virus (HIV) prevention and treatment globally; however, HIV and/or acquired immunodeficiency syndrome (AIDS) management for children appears to always lag behind the progress made for adults. South Africa operates the most extensive HIV treatment programme in sub-Saharan Africa. Even with greater availability of antiretroviral therapy (ART), controlling the infection continues to be difficult. To reduce HIV-related deaths, retention on ART and viral suppression are crucial. Although advances in ART medication development provide great hope, improvement in care is still needed. In this review, challenges related to ART for children and adolescents living with HIV are highlighted.

**Aim:**

To review the challenges experienced in the drug management of children and adolescent patients on antiretroviral therapy globally.

**Method:**

An extensive online database search was conducted using keywords on Google Scholar, PubMed, Medline, ISI Web and Embase in February 2025, for English language literature published between 2015 and 2024 in the public healthcare sector. All studies (*N* = 33) that met the inclusion criteria and reported on challenges experienced by children and adolescents living with HIV from prematurity to 15 years, their caregivers, and prescribers of HIV treatment were retrieved and reviewed.

**Results:**

Challenges found in the management of HIV in children and adolescents included adherence monitoring, medicine-related factors, psychological factors, social factors and health system-related factors.

**Conclusion:**

Challenges still exist in the management of children and adolescents living with HIV, despite the great strides made. Addressing these challenges is imperative and necessitates a multifaceted approach involving various interventions.

**Contribution:**

The findings of this study emphasise the need for a holistic approach to addressing the identified challenges in order to improve treatment outcomes.

## Introduction

The sub-Saharan African (SSA) region remains the most severely impacted region globally by the human immunodeficiency virus (HIV) epidemic. Notwithstanding the fact that SSA has just over 10% of the world’s population, it accounted for 65% of all the people living with HIV in 2021.^1^ Although Vertical Transmission Prevention initiatives have reduced the rate to 3% and improved the early infant diagnosis rate to 93% in South Africa in 2022,^2^ children aged 0–14 still lag behind adults in achieving the 95-95-95 targets.^3^ In 2022, the SA adult population reached a 94%-80%-92% adherence to these targets, while children fell short at 82%-65%-67%.^2^

Access to antiretroviral therapy (ART) has increased remarkably in SSA, with the World Health Organization (WHO) estimates pointing to an increase from 100 000 people at the end of 2003 to over 2 million in December 2007, representing a 20-fold increase.^1^ This advancement transformed the clinical course of HIV. However, a new challenge emerged, shifting the focus from medication availability and access to effective HIV management. This responsibility now extends beyond health care professionals to patients and caregivers, whose engagement in treatment has become integral to obtaining positive health outcomes.^1,2^

By the end of 2023, approximately 39.9 million people were living with HIV, including 1.4 million children (aged 0–14 years) and 38.6 million adults (aged 15 years and above). Antiretroviral therapy coverage was 77% among adults and 57% among children under 15 years.^1^

Human immunodeficiency virus, being a chronic disease, means life-long therapy, sometimes with complex regimens, resulting in increased side effects compared to those experienced with simple regimens.^4^

In 2023, 90% of the 1.4 million children living with HIV were in SSA.^1^ Acquired immunodeficiency syndrome (AIDS) still ranks as the second leading cause of death among adolescents worldwide, even though ART is freely available and has contributed to the global decrease in AIDS related deaths.^5^ In 2020, 320 000 of an estimated 680 000 people dying from HIV-related illnesses were adolescents aged 10–19 years.^5^

At the 2015 United Nations’ Summit, WHO amended the age and medical restrictions for ART initiation,^6^ recommending that all individuals who were infected with HIV should start ART immediately, making all populations and ages eligible for treatment. All HIV-exposed infants received ART prophylaxis. As these guidelines were implemented, access to ART for children and adolescents scaled up worldwide, with many more infants, children and adolescents taking antiretroviral medications.^6^

Antiretroviral therapy has undergone major changes from monotherapy to dual therapy, and now triple therapy, leading to an increased number of medicines to take. In recent years, greater focus has been placed on better-tolerated combinations and formulations.^7^ However, this has not been forthcoming for paediatric medicines. Similar to most disciplines in medicine, there is always a delay in the development of appropriate paediatric ART formulations with adult research taking preference,^7^ hence posing a challenge for the management of HIV in children.

Despite many innovations, limited paediatric formulations are still prevalent compared with adult formulations.^8^ Without adequate or convenient paediatric formulations, prescribers and caregivers resort to cutting, crushing or substituting adult medications to meet the need for easier administration of medications for children.^8^ This can have drastic, negative impacts on the children’s management, leading to over- or under-dosing of the ARV medicine.^8^ Some of the medicines are not palatable, leading to poor intake of the medicine by children, among other challenges.^8^

Adherence to ART is crucial to HIV management, as it leads to lower viral loads, decreased symptoms in patients, and decreased viral resistance.^9^ Viral resistance to first-line ART requires patients to switch to more expensive and less available second- and third-line therapies.^9^ With good ART adherence, children and adolescents living with HIV can live long, healthy lives; therefore, it is a priority to study and address the causes of poor adherence with the goal of maintaining successful therapy for as long as possible. Assessing and monitoring adherence with ART is recommended in routine practice as part of management programmes,^10^ however, it is difficult to precisely assess and monitor. Various assessment methods are available, but they have varying degrees of validity and reliability (e.g. self-reporting by patient and/or caregiver, directly observed therapy, appointment keeping, clinically tested and recorded virological response and pharmacy pill-counting). The virological response to treatment is the most effective means to assess treatment adherence in patients on antiretroviral therapy and therefore validates adherence assessment using other methods.^11^

Further, children are at particularly high risk of poor adherence as they depend on a responsible caregiver or parent for their medication to be administered accurately.^11^ Also, children differ from adults in many aspects of pharmacotherapy, including medicine-related toxicity, taste preferences and capabilities of drug administration.^12^ Therefore, the ARV medications need to be ‘child-friendly’ or highly acceptable in terms of dispensability, palatability, swallowability, ease of transportation and storage, to facilitate adherence of the children to their medications, which in turn will ease the burden on the parent or caregiver.^12^

Drug design and manufacture go beyond the standard principles of producing a drug with the best therapeutic profile.^13^ It is crucial that paediatric medications are formulated to best suit a child’s size, age, physiological condition and treatment requirements.^13^ Drug design and manufacture must therefore involve the manufacture of drugs that are highly acceptable to the target age group, in this case, the paediatric population, in terms of taste (sweet, sour, bitter, salty, etc), texture, flavour, colour, shape, size, composition, dosage form, dosing frequency and quantity.^13^ To ensure adequate treatment of all children, different routes of administration, dosage forms and strengths may be required.^13^

Some of the formulations used in South Africa (see Table 1) are complex and not child-friendly, which often leads to off-label and unlicensed use of adult medicines for children.^13^

**TABLE 1:** List of antiretroviral formulations available for use for children in South Africa.

Single agents	Fixed drug combinations (FDCs)
**Liquid form** Abacavir (ABC) Lamivudine (3TC) Nevirapine (NVP) Zidovudine (AZT)	**Liquid form** Lopinavir/ritonavir (LPV/r)
**Tablet form** Abacavir (ABC) Lamivudine (3TC) Efavirenz (EFV) Dolutegravir (DTG) Ritonavir (RTV)	**Tablet form** Lopinavir/ritonavir (LPV/r) Darunavir/ritonavir (DRV/r) Atazanavir/ritonavir (ATV/r) Abacavir/lamivudine (ABC/3TC) Emtricitabine/tenofovir (FTC/TDF) Lamivudine/dolutegravir (3TC/DTG) Tenofovir/Emtricitabine/Efavirenz (TDF/FTC/EFV) Tenofovir/lamivudine/Dolutegravir (TDF/3TC/DTG)
**Dispersible tablet form** Dolutegravir (DTG)	**Dispersible tablet form** Abacavir/lamivudine (ABC/3TC) Zidovudine/lamivudine (AZT/3TC)
**Capsule form** Efavirenz (EFV) Atazanavir (ATV)	**Pallet form** Lopinavir/ritonavir (LPV/r)
**Powder form** Ritonavir (RTV)	
**Injection form** Zidovudine (AZT)	

ARV, antiretroviral.

Formulation acceptability and preferences facilitate medication adherence in children, and they are important factors in achieving the intended treatment outcomes.^14^

Some capsules and tablets cannot be swallowed whole by young children because of size, unless manipulated. Manipulation of drugs could result in either underdosing or overdosing of the medication, leading to sub-therapeutic doses or toxicity, which invariably affects positive health outcomes.^15^ Developing child-friendly formulations of ART will make it easier for caregivers to administer medications to children and easier for children to take the medications. Both strategies result in positive health outcomes. Therefore, identification of the difficulties experienced by caregivers in the process of administering ART formulations to children is essential for better management of children and adolescents living with HIV, and to sensitise manufacturers and policymakers to introduce child-friendly formulations into the marketplace.

This review will investigate and highlight the challenges experienced in the drug treatment of children and adolescents living with HIV globally.

## Methods

A narrative review was preferred and found to be appropriate since it provides a synthesis of available knowledge about drug management challenges faced by children and adolescents on ART. It further provides a hierarchy of evidence, which is highly useful to medical researchers. The availability of ART has changed the prognosis of HIV infection, but it is concerning that many children still fail to thrive. This narrative review sought to better understand the problem by exploring what studies in different contexts could be found.

A comprehensive electronic structured search for English language literature was conducted on 20 February 2025 using Google Scholar, PubMed, Medline, ISI Web and Embase as search engines. The keywords entered either on their own or in combination to create Boolean phrases were: Challenges, drug, management, paediatric, patients, antiretroviral, treatment, globally, Africa and South Africa. On Google Scholar, the phrase ‘challenges with paediatric antiretroviral drug management’, with a customised timeframe of 2015 to 2024 was used. On PubMed, the search criteria implemented were (antiretroviral therapy [Title and Abstract] OR ART drug management challenges [Title and Abstract]). MeSH terms included: Antiretroviral therapy; drug management; paediatric; challenges. A qualitative appraisal of the studies was conducted, but no formal scoring of the study quality was done.

### Inclusion criteria

All studies that reported on challenges experienced by children and adolescents living with HIV, caregivers and prescribers in HIV management from the age of prematurity to 15 years in public healthcare settings globally were included. In South Africa, the vast majority of children and adolescents living with HIV receive their antiretroviral therapy (ART) through the public healthcare sector. This sector operates the world’s largest public ART programme, providing essential treatment to most of the young people affected by HIV. Although a small minority may access private healthcare, the public system remains the primary source of ART for this population, and it is free. Therefore, the variation of responses from children and their caregivers in the public sector is believed to be representative of the challenges that may be experienced in the private sector. In addition, since the research was going to be conducted in the public sector, it was decided to look at public sector original articles so comparisons can be valid. Only original research studies that were published from January 2015 to December 2024 were included.

### Exclusion criteria

Reports from private healthcare settings were excluded. Surveys and opinion pieces which were not based on primary research were also excluded, as were studies published in languages other than English. Surveys were excluded because of the lack of clear data collection methods that do not allow for the generalisation of the results.

### Reliability and validity of data extraction and processing

A data charting list was created and utilised to collect relevant information. Details such as publication year, study design, country of origin, team composition and outcomes were gathered. Data were recorded at each stage, and the three authors conferred to review the findings. Any issues that arose were addressed by consensus, ensuring the reliability, validity and reproducibility of the results.

**FIGURE 1:**
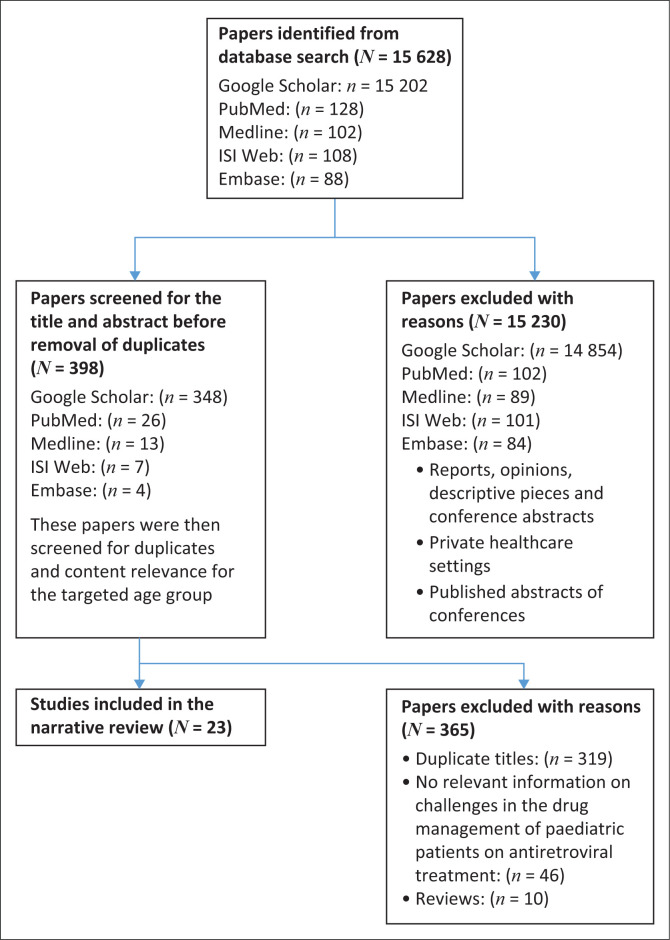
Flow diagram showing the study selection process.

Figure 1 illustrates the process undertaken to select the relevant literature for the study.

The risk of bias was assessed by checking how the data were collected. This included non-response bias, recall bias, proxy response bias, language bias, fatigue bias and timing bias, making the study more transparent and reproducible. For recall bias, the articles were checked for the response rates from participants, and reasons for non-participation were also checked and observed if recorded. For recall bias, all retrospective studies were checked for self-reported information and how the studies attempted to minimise the bias. For proxy response bias, where the caregivers responded on behalf of children, the studies were checked if this was acknowledged as a limitation. For language bias, the studies were checked if the data collection instruments or interviews were conducted in the participants’ preferred language. Information on the language(s) used, translation processes and possible inclusion or exclusion of non-native speakers. Observation was done to check if the studies captured this as a limitation if no translation was done, and if non-native speakers were excluded. For fatigue bias, the studies were assessed on the length and possible complexity of interviews and/or surveys and whether the study mentions breaks or measures to mitigate fatigue. For timing bias, details about the timing of data collection and whether the authors discuss its potential impact were checked from the studies included.

### Synthesis of how study quality may affect conclusions

The validity and reliability of the data are essential since this review has used the studies to develop the conclusions. Assessing the strengths and weaknesses of included studies is essential. Therefore, reporting how each potential bias was assessed and its potential impact on findings increases transparency. This, in turn, allows other researchers to reproduce the assessment and understand the trustworthiness of the evidence.

### Ethical considerations

Ethics approval was not required for this study as the data are freely available in the public domain. This article followed all ethical standards for research without direct contact with human or animal participants, and all studies included had acquired ethical clearance.

## Results

Many challenges have been reported (as depicted in Table 2), and they have been tabulated into types, the number of studies that have cited this as a challenge, the country where the research was done, together with the year, reference and author details.

**TABLE 2:** Challenges experienced in the drug management of children and adolescents living with human immunodeficiency virus.

Number	Type of challenge	Number of studies cited	Countries/regions/continents	Year	Reference numbers	Author
**1**	**Adherence testing and monitoring**	5	US	2020	19	Berendam et al.
			Lesotho	2018	16	Amzel et al.
			Democratic Republic of Congo	2020	17	Moudachiro et al.
			Colombia	2023	38	Lopez De La Espriella
			Namibia	2024	18	Munyayi & Van Wyk
**2**	**Medication-related factors**					
	Formulation issue	10	Thailand	2015	24	Kang et al.
			US	2019	25	Willis et al.
			Switzerland US Spain South Africa Kenya	2022	20	Penazzato et al.
			India	2023	23	Garg et al.
			UK	2024	48	Bamford et al.
			US UK	2024	21	Buchanan et al.
			Netherlands UK Spain Thailand France Senegal Cameroon Uganda Zimbabwe South Africa	2024	22	Kamphius et al.
			South Africa	2024	26	Okafor
			Kenya	2024	28	Sliefert et al.
			India	2024	27	Kapoor et al.
**3**	**Side effects**	4	Thailand	2015	24	Kang et al.
			US	2019	25	Willis et al.
			Nigeria	2021	40	Busari et al.
			Colombia	2023	38	Lopez De La Espriella
**4**	**Concomitant use of alternative medicines**	2	South Africa	2016	35	Loeliger et al.
			Ghana	2022	34	Atanuriba et al.
			Burkina Faso	2017	36	Dahourou and Leroy
			Kenya	2018	29	Pilgrim et al.
			Ghana	2021	37	Klutsey et al.
			Colombia	2023	38	Lopez De La Espriella
			Namibia	2024	18	Munyayi and Van Wyk
			US	2019	25	Willis et al.
			Kenya	2024	28	Sliefert et al.
			Ghana	2021	37	Klutsey et al.
			Nigeria	2021	40	Busari et al.
			Colombia	2023	38	Lopez De La Espriella
			Namibia	2024	18	Munyayi and Van Wyk
**5**	**Socio-economic issues**	2				
			US	2019	25	Willis et al.
Colombia	2023	38	Lopez De La Espriella
**6**	**Health system inefficiencies**					
	Access to care	1	US	2019	25	Willis et al.
	Quality of care	8	South Africa	2018	42	Williams et al.
			US	2019	25	Willis et al.
			South Africa	2021	46	Myburgh et al.
			Ethiopia	2021	49	Gelaw et al.
			Australia (in SSA)	2018	45	Karimina et al.
			US	2023	39	Rosen et al.
			Namibia	2024	18	Munyayi and Van Wyk
			South Africa	2024	26	Okafor

Note: Please see the full reference list of this article, Mthethwa PP, Naidoo P, Taylor M. Challenges with drug management of children and adolescents living with human immunodeficiency virus on antiretroviral therapy: A narrative review. Afr J Prm Health Care Fam Med. 2026;18(1), a5121. https://doi.org/10.4102/phcfm.v18i1.5121, for more information.

HIV, human immunodeficiency virus; SSA, sub-Saharan African; US, United States; UK, United Kingdom.

The type of challenge from Table 2 was further expanded to give more detail, as seen in Table 3.

**TABLE 3:** Challenges explained according to identified themes.

Number	Type and list of challenges
**1**	**Adherence testing and monitoring**
	Periodic viral load testing and monitoring not effectively done
**2**	**Medication-related issues**
2.1	Formulation issues
	Lack of appropriate formulations and dosage forms specific for children, including premature infants and neonates
	Inaccurate weight-based dosing
	High pill burden and lack of FDC preparations for children
	Complex solid preparation and administration (including measuring, crushing, dissolving, mixing) and frequent inability to swallow pills or tablets
	Drug storage requirements for liquid medication
	Inability to maintain privacy and confidentiality, especially during times of travel, because of complex preparations with inappropriate dosing schedules
	Bitter taste of syrups resulting in poor palatability
	Poor solubility of drugs
2.2	Side effects
	Side effects experienced by patients leading to poor adherence to ART: lipodystrophy, dyslipidaemia, disorders of glucose metabolism, as well as osteopenia and renal dysfunction
2.3	Concomitant use of alternative medicines
	Concomitant use of traditional, complementary and alternative medicine
**3**	**Caregiver issues**
	Non-disclosure of HIV status to children
	Caregiver’s fear of the child’s reaction to disclosure
	Dependence of infants and children on others for medication administration
	Caregivers confronted with a child’s refusal to take treatment
	Caregivers may be occupied with their own health issues
	Caregiver’s anxiety and depression resulting from non-acceptance of their own HIV status
	Inadequate psychological preparedness of the caregiver to support the children’s adherence
	Challenge of multiple caregivers
	Non-disclosure of the child’s HIV status to other family members because of fear of stigmatisation
	Little or no interest of caregivers in being engaged in their children’s health care
	Continuity of child care compromised because of frequent switching of caregivers for children in orphanages.
	Caregivers not finding time to collect treatment because of work issues
**4**	**Psychosocial factors**
	Forgetfulness of adolescents to take medication because of a change in daily routine or when far from home.
	Fear of stigmatisation of status disclosure
	Fear of discrimination
	Anxiety and depression resulting from the non-acceptance of the HIV status by the child
	Psychological, emotion and physical changes associated with adolescents
	Lack of motivation or desire to take one’s own treatment, and long-term treatment-induced exhaustion by adolescents
**5**	**Socio-economic factors**
	Unemployment and the low socio-economic status of the caregiver
	Association of the level of education with the misunderstanding of disease transmission and treatment
	Long distance travelled to the health facility
**6**	**Health system inefficiencies**
6.1	Access to care
	Challenges with accessibility of treatment
	Lack of health facilities providing HIV/AIDS services in local communities
	Lack of Differentiated Service Delivery (DSD) models, including adherence clubs (ADs), on sustaining retention in care
6.2	Quality of care
	Medicine shortages
	Long waiting times and poor staff attitude
	Staff shortages
	Lack of training of staff on HIV management
	Lack of competent managers for multidisciplinary collaboration to guarantee optimal treatment for all patients
	Poorly managed workplace conflicts wreak havoc on work productivity, demoralise staff, increase turnover and damage relationships in the workplace
	Medical doctors not interested in or comfortable with dealing with children
	Prior exposure to treatment in the public/private sector and vice versa
	A lack of coordination between paediatric and adult services.
	Poor quality of patient-prescriber relationship affects the decisions that clients make regarding their care, causing loss to follow-up (LTFU)
	LTFU associated with sex, age, rural settings and issues of malnutrition
	Drug resistance and treatment failure

FDC, Fixed drug combinations; HIV/AIDS, human immunodeficiency virus/acquired immunodeficiency syndrome; ART, antiretroviral therapy.

## Discussion

A total of 23 studies were relevant to the title of the review. The six most commonly reported categories of challenges were: Adherence testing and monitoring (*n* = 5), medication-related issues (*n* = 14), caregiver issues (*n* = 5), psychosocial issues (*n* = 6), socio-economic issues (*n* = 2) and health system inefficiencies (*n* = 9).

Studies on challenges relating to medication-related issues, caregiver issues, psychosocial issues, as well as quality of care issues appear across multiple countries, while those on adherence monitoring, access to care and socio-economic issues seem more limited.

### Adherence testing and monitoring

In the last 4 years (2020 to 2024), studies on adherence monitoring in children have been conducted in North America, South America, Asia and Africa.

Various methods available to measure and monitor adherence to HIV treatment included viral load testing, pharmacy pill counts and monitoring the frequency of missed appointments to collect medication. Guidelines indicated the type of methods that could be used as well as the frequency at which they can be monitored. However, viral load testing and monitoring were shown not to be effectively done at the required intervals in low- to middle-income countries (LMICs).^16,17,18^

### Medication-related issues

#### Formulation issues

Many studies have been conducted across four continents (Africa, Asia, Europe and North America). These studies have identified formulation issues as a major challenge to the effective management of HIV in children.

The current selection of antiretroviral (ARV) drugs that can be used for initial therapy in children living with HIV is very limited.^19^ This limitation is even more pronounced in formulations suitable for premature infants and neonates.^20^

Neonates undergo rapid weight changes and physiological processes, which impact their drug absorption, distribution, metabolism and excretion (ADME) compared to older children.^21,22^

Formulation challenges affect countries globally, as cited in the studies accessed. A number of the studies reviewed in this article cited challenges with a lack of appropriate formulations and dosage forms for children^21,23,24^ and neonates. These challenges were especially noted for those born prematurely, of low birthweight, and for potential use during breastfeeding.^22^

The high pill burden because of the use of single agents causes challenges with adherence to treatment.^25^ Some drugs require once-daily dosing while others require twice-daily dosing within the same treatment regimen. The different dosing schedules for these individual drugs within the same regimen sometimes causes confusion for the caregivers and adolescent children taking their own medication.^24^ Certain regimens contain different dosage forms (tablets and syrups). This creates problems for caregivers as some children may be able to tolerate one dosage form and not the other, and may refuse to take medication from the undesirable dosage form.^24^

In addition, there is a frequent inability to swallow tablets because of size and quantity.^26,27^ This requires caregivers to correctly measure and break tablets, which is difficult if the tablets are not scored, resulting in inappropriate doses being given to children. Others may, therefore, require crushing and dissolving in liquids before administration.^24^

Another concern is an inappropriate dosing schedule. Caregivers are unable to maintain privacy and confidentiality, especially during times of travel, because of the need to crush tablets, mix powders with water and other liquids, in preparation of medication by caregivers for administering to the children, especially in cases where the status of the child is undisclosed.^28^

Previous studies showed that poor palatability is directly associated with poor adherence resulting from the bitter taste of the liquid formulations associated with protease inhibitors (PI).^8,25,28^ Although some patients have been switched to paediatric DTG-based regimens associated with better taste, there are some patients who are not yet eligible for switching to DTG and still remain on the bitter-tasting liquid formulations.

There are challenges with weight-based dosing in infants and younger children associated with the frequent changes in weight of infants and children,^21^ requiring frequent visits to health facilities for dose adjustments.

The additional storage requirements for liquid medication pose challenges for caregivers who do not own refrigerators, having to find alternative ways to maintain the temperature requirements for the liquid preparations.^29^ This creates an even bigger challenge if the status of the child has not been disclosed to other family members within the same household.^29^

#### Side effects

In this review, four of the 33 studies cited side effects as one of the factors that pose a challenge in the management of children living with HIV, as it significantly affects adherence to treatment. The studies cited were conducted in five of the seven continents (Africa, North America, South America, Asia and Europe). Side effects such as lipodystrophy, dyslipidaemia, disorders of glucose metabolism, osteopenia and renal dysfunction posed major challenges to managing the HIV infected children.^24^

In a more recent study by Sindie et al., it was evident that gastrointestinal symptoms are most frequently experienced by children on ART.^30^ Zidovudine, lopinavir/ritonavir, efavirenz and nevirapine-based regimes are significantly associated with haematological, gastrointestinal, neurological and dermatological adverse drug reactions (ADRs), respectively. Children with immunological suppression are at a higher risk of developing ADRs compared to those without this.^30^

A successful 2021 trial introduced and rolled out the 10 mg scored and dispersible dolutegravir (DTG) tablet for children, as DTG-based ART was shown to be superior to other ART regimens for children, such as ritonavir-boosted lopinavir (LPV/r) and efavirenz (EFV)-based ART, with a lower side effect profile.^31^ Side effects include headache, nausea and vomiting, and diarrhoea. Serious side effects include allergic reactions, liver problems, neuropsychiatric symptoms and weight gain. Globally, HIV/AIDS treatment guidelines have been changed to integrase strand transfer inhibitor (INSTI) based regimens, with DTG most used globally, since 143 countries, including 41 LMICs, have included DTG into first-line treatment recommendations for all ages.^32^

Dolutegravir has the most neuropsychiatric side effects compared to all other INSTIs. Weight gain was reported with all INSTIs, especially with DTG, with differential effects according to sex and ethnicity, mostly affecting female and non-white patients.^33^

#### Concomitant use of alternative medicines

The concomitant use of traditional, complementary and alternative medicine (TCAM) in the treatment of HIV infection is quite common in low- and middle-income countries, including South Africa.^34^ Optimal adherence to ART is key to sustained viral suppression, and a great concern has been whether the concomitant use of TCAM has an impact on adherence to ART. The Loeliger et al. study proposed a strengthening of collaboration between community health care providers and traditional healthcare providers as a means of sustaining adherence to ART.^35^

### Caregiver-related issues

Studies carried out in the last 8 years in Africa and South America showed the significant role played by caregivers in ensuring that young children adhere to treatment, thereby improving immunity and viral suppression to HIV, thus improving overall treatment outcomes. Treatment of young children (usually younger than 12 years) depends on a caregiver (parents or other family members or anyone else). Treatment success, therefore, relies on the caregiver, who needs to be highly involved, motivated and concerned with adherence issues.

In a 2017 study by Dahourou & Leroy in West Africa, it was evident that a number of caregiver factors influence the level of adherence to treatment.^36^

Being the caregiver of a child on ART comes with different types of neurological effects, such as anxiety.

Caregivers’ fear of disclosing the status of the children to them, because of uncertainty of how the disclosure will be handled by the child, is because of their fear of poor adherence to treatment and negative psychological effects on the children.^37^ In a study conducted in Ghana, the fear of caregivers was also associated with whether the children had the required maturity to understand and accept their HIV status and also have the ability to be discreet about their HIV status outside their households, such as in public and social spaces, at school and with friends. This fear is associated with the possible stigmatisation and discrimination that could be attached to it.^37^ The same results were observed from a study in Ghana in recent years.^37^

The non-disclosure of HIV status to the child by the caregiver negatively affects adherence to treatment.^27^ Caregivers may, therefore, also be confronted with the child’s refusal to take the treatment, as a result of not understanding the importance of taking treatment, owing to non-disclosure, resulting in a number of frequently missed doses, significantly affecting adherence.^37^ A study conducted recently in 2023 in Colombia on children under 13 years of age, revealed that the majority of the children were unaware of their diagnosis (86%), which contributed to non-compliance, because of their not understanding the importance of taking medication daily.^38^

In a study conducted in Africa in 2021 by Klutsey et al., adherence was seen to be poor because of non-disclosure. This results from the fear of the child’s reaction anticipated by the caregiver, causing the caregiver to not disclose the status to the child.^37^

The caregiver’s own busy schedule, including issues with their own positive HIV status and general health, may result in disengagement, treatment oversight and forgetfulness to give the medication to the child.^36,37^ The caregiver’s concern over their own HIV status and acceptance thereof may result in anxiety and depression. This often leads to alcohol and substance abuse by the caregiver, affecting their ability to take care of the child.^37^

In a study conducted in Zimbabwe, inadequate caregiver psychological preparedness to support children’s adherence has also been shown to negatively affect adherence.^25^ This resulted in the caregiver not fully understanding the importance of administering the medication to the children and forgetting to administer medication to younger children who do not yet have the mental ability to remind themselves to take medication.^25^

A study done in Burkina Faso (in West Africa) revealed that children with several caregivers did not adhere to treatment, as well as those with a single caregiver.^36^ Children whose mothers were the primary caregivers showed better treatment responses compared to those cared for by others.^36^ This effect was even more pronounced when the mother was also receiving ART. These findings may reflect the strong emotional bond between mother and child, or possibly the mother’s feeling of guilt about her child’s infection.^36^ This feeling would thus protect the child from poor adherence.^36^

The fear of being stigmatised by members of the family or the community is believed to be associated with poor adherence^29^ leading to non-disclosure of HIV status to other family members.^29^ The non-disclosure often leads to the parent and/or caregiver feeling and receiving inadequate support from other family and/or household members as a result of their HIV status not being known to them.^39^ Fear of stigmatisation is still frequently observed in Africa, and some mothers take care of their child’s treatment without the father knowing, or they do not want their child to be treated out of fear of letting the father know about the child’s HIV status, and thus the father’s infection status.^29^

The caregiver’s non-disclosure of HIV status to children who are age-eligible for disclosure, and little engagement of caregivers in the children’s care, was also observed to negatively affect adherence. Some caregivers had little or no interest in being engaged in their children’s health care and therefore, neither brought the child to their appointment nor ensured if the child took their medication.^29^

The continuity of child care may also be compromised because of frequent switching of caregivers for children in orphanages and care centres, with the new caregiver being unaware of the child’s HIV status.^29^ The caregiver may also not be able to find time to collect treatment for the child timeously because of his/her work issues.^36^

### Psychosocial issues

A significant relationship exists between the psychological and social factors and patient adherence to ART. To date, the fear of stigmatisation of people living with HIV negatively affects adherence to treatment.^25^ In SSA, the perpetuation of stigma because of cultural beliefs and misinformation about HIV infection itself remains a great challenge.^25^

The transition from childhood to adolescence is a critical stage in HIV management, marked by significant physical, mental and emotional changes. Children should be informed of their HIV status before this stage so that they can take responsibility for their own health.^36^ Human immunodeficiency virus status awareness leads to better adherence^38^ However, if the information is not transmitted adequately to the adolescent, it might lead to poor adherence because of the resulting anxiety and depression.^40^ This phenomenon is more prevalent in adolescents than in younger children.^39^ It is then difficult to determine whether the observed poor adherence is because of their being adolescents or to challenges with ART.

A Namibian study conducted in 2024 showed that virological suppression in adolescents up to the age of 15 years was lagging behind compared to younger children and adults.^18^ This was attributed to adolescent-associated factors such as their sensitivity to peer pressure, causing adolescents to involve themselves in undesirable social activities (alcohol and substance abuse),^41^ thus negatively affecting adherence.

Depressed adolescents tend to miss doses more frequently because of forgetfulness and the frequent need to take medication in private spaces without being seen,^40^ which is closely associated with the fear of discrimination and stigmatisation, leading to non-adherence.^40^ The severity of the depression experienced by adolescents ultimately leads to alcohol and substance abuse.^18^

When adolescents take their own treatment, they may forget doses^40^ because of changes in their daily routine. Lack of desire to take one’s own treatment, and long-term treatment-induced exhaustion are also determining factors of poor adherence at the adolescent age.^36^

### Socio-economic issues

Two studies cited socio-economic factors as barriers to adherence. A poor socio-economic status (assessed by the absence of television (TV), electricity, food, a fridge at home and by the inability to pay transportation fees to come for medical visits) was described as a determinant of poor adherence,^25,38^ while improvement in socio-economic status was reported to favour virological success.^25^

The economic status of caregivers plays an important role in adherence. Many studies reported that factors, such as the long distance from home to the health facility, the availability and mode of transport to the health facility, including poor weather patterns, contribute to caregivers not collecting medication from the health facility.^17,40^

A study conducted in South America also showed that the low socio-economic status of caregivers increased non-adherence to treatment. The majority of caregivers (90.5%) earning less than the minimum wage were seen to have poor adherence.^38^

A South African study reported that the caregiver’s high level of education was associated with better adherence.^38^ The level of understanding of the disease itself, however, impacts the transmission and treatment, leading to poor health-seeking behaviours and low adherence to treatment by the caregiver.^38^

### Health system inefficiencies

#### Access to care

Limited access to treatment affects adherence to treatment.^42^ This is a concern given the already lagging ART coverage in children compared to that of adults.^39^

Studies showed that patients travel long distances to access health services. This results from the lack of health facilities in some of the local communities, forcing patients to travel long distances to access health services.^42^

Differentiated Service Delivery (DSD) models, such as adherence clubs (ACs), are client-centred approaches where clinically stable patients collect their medication. When implemented, ACs decrease congestion at health facilities and are proven to sustain retention in HIV care and adherence to ART.^42^ The limited availability or lack thereof hinders reducing the congestion at health facilities, which enables the decrease in long waiting times in congested facilities.

#### Quality of care

**Medicine shortages:** A health system-related factor that negatively affects adherence is medicine stock-outs. Many countries in Africa, such as Kenya, Uganda and South Africa, often face stock outages, which affect adherence. Stock-outs delay treatment initiation, cause incomplete treatments, treatment interruptions, reduce patient engagement and increase costs. This raises the risks of drug resistance and treatment failure.^40^ Hwang et al. studied the number of self-reported ART stock-outs among medication dispensers in South African health facilities.^43^ While there were limited reports of paediatric ART stock-outs in the study, the stocks-outs affected adherence in the patients.^43^

Availability of paediatric formulations is likely to be more problematic than that of adult formulations because of the potential need for cold storage, short shelf lives, and lower demand. Factors that contribute to stock-outs of ART in SSA include inefficiencies in drug supply, dependence on international aid contributions, poor coordination with port authorities, and inadequate government funding.^40^

**Long waiting times and poor staff attitude:** In SSA, the long waiting times at health facilities affect adherence as patients are not prepared to wait. They leave the facility without collecting the medicines and sometimes do not come to collect their medication at all.^25^

#### Human resources issues

**Staff shortages and lack of training of staff:** A multidisciplinary team appropriately manages HIV patients. There are considerable challenges related to the shortages of clinical healthcare practitioners (doctors, nurses, pharmacists & social workers) needed for the provision of comprehensive ART services at primary health care (PHC) health facilities. This compromises the quality of services received by patients at some of the health facilities.^42^

Some staff members are not interested in managing children as they regard them as being too complex to manage.^42^

Studies also showed that a lack of doctors visiting PHC clinics from hospitals, a lack of periodic training programmes on the provision of ART to children, and minimal on-site mentorship of professional staff regarding HIV disease in children, negatively affect the provision of quality service to patients, ultimately affecting treatment outcomes.^42^

William et al. showed that a lack of competent managers to ensure multidisciplinary collaboration affects treatment outcomes.^42^ They also revealed that poorly managed workplace conflicts cause havoc on work productivity, demoralise staff, damage relationships and ultimately increase turnover.^42^

#### Prior exposure to antiretroviral treatment

An important aspect of effective management and preventing treatment failure is accurately understanding the patient’s ARV prior exposure history before they enter a treatment programme.^44^

Generally, there is evidence of self-reporting, particularly for women of child-bearing age, but there is no evidence of self-reports from other categories of patients. Hair or plasma of individuals who self-reported that they have no prior exposure to ART upon treatment initiation were used to test ART drug concentration.^44^ Eight studies done prior to 2021 reported on the use of hair or plasma as objective and accurate methods to estimate adherence and sustained drug concentrations in patients.^44^ However, these methods are expensive, and their usage is therefore limited.^44^

#### Patient loss to follow-up (loss to follow-up)

Insufficient coordination between paediatric and adult services made it difficult for adolescents to navigate the health system during their transition to adult care, leading to substantial loss to follow-up among this group.^45^ Limited data are available on the relationship between the patients and the prescribers and how it impacts adherence and LTFU. Poor quality of patient-prescriber relationship affects the decisions that clients make regarding their care, e.g. collecting treatment on time from the facility, because of the type of patient experience of care received by patients at health facilities.^46^

Cohort data from the Asia-Pacific, the Caribbean and Central and South American regions have found LTFU associated with sex, age and rural settings, while data from Thailand in the Asian-Pacific region also factored in issues of malnutrition because of the low socio-economic status of patients, impacting their ability to take medication.^45^

#### Drug resistance and treatment failure

Dolutegravir has the ability to facilitate co-treatment of children with HIV and tuberculosis co-infection, as the interaction with rifampicin can be managed by administering DTG twice daily, doubling the daily dose.^47^ However, the possible development of drug resistance to DTG in children, which may result from previous exposure, remains a concern in maintaining children on first-line treatment.^48^

Two studies in Sub-Saharan Africa cited treatment failure as a challenge in paediatric ART management. Treatment failure was found to be significantly higher in SSA compared to other continents.^49^ Factors that were significantly associated with treatment failure among children living with HIV were age below 5 years, children with a history of opportunistic infections, advanced WHO clinical stage, children with previously interrupted and re-started treatment, original regimen change and ART drug substitution. Thus, emphasis should be given to prevent opportunistic infection and improve treatment adherence level, which in turn helps to tackle HIV drug resistance and keep the patient on first-line regimens.^48,49^

### Limitations

This study presents some limitations. Only papers published in English were considered for this narrative review. The restriction to only English language studies could have excluded some relevant studies. There may have been papers published in other official languages that reported on challenges with ARV drug management of children that could have been excluded. The study also excluded papers conducted in the private sector. There may have been challenges identified in the private sector that have not yet been experienced in the public sector, which could have limited our results.

This being a narrative review, the study lacked the rigour and structured methodology, offering a broad synthesis which could be more subjective and making the study prone to bias. However, the evaluation of the quality and/or risk-of-bias was done by checking how the data were collected. This included non-response bias, recall bias, proxy response bias, language bias, fatigue bias and timing bias. It is, however, unlikely that these limitations affected the interpretation or conclusions of this study.

Although the selection of published studies guards against the selection of studies that may not have undergone intensive review processes, where the reproducibility of the data can be guaranteed, it also limits data that could have been included, which may not yet be published.

The focus on public sector care may limit the perspectives of participants in the private sector, which may be different.

### Recommendations

#### Future research

Special focus and attention should be given to making more child-friendly formulations available by pharmaceutical companies in the shortest timeframe possible.

#### Clinical practice

Clinicians and programme managers should make specific short-term interventions for adolescents living with HIV, focusing on stigma reduction, disclosure challenges and coping mechanisms for ART. These interventions should be shared with both healthcare professionals as well as patients and their caregivers through training.

Preventing opportunistic infections and enhancing ART adherence levels are crucial approaches to improving treatment outcomes and eliminating treatment failure.

#### Policymakers

Policymakers should put continuous efforts towards finding innovative ways to limit and ultimately eliminate loss to follow-up of patients and retain patients on ART.

## Conclusion

This review has highlighted and confirmed that challenges that affect the successful management of children and adolescents living with HIV do exist. These challenges include various factors such as inadequate adherence monitoring, medicine-related factors, psychological factors, social factors, as well as health facility-related factors.

### Policymakers/future research

Human immunodeficiency virus affects many people in many countries. The pharmaceutical response to save lives has been successful concerning adults, but more needs to be done to address the HIV treatment of children by pharmaceutical manufacturers, governments, through financing adequate treatment for children and adolescents living with HIV, by improving the Health Systems and efficient access to ART, and addressing the social services and support required by caregivers.

### Multi-sectoral collaboration

Communities, including churches and other religious organisations and schools, should be active concerning the stigma associated with HIV infection. Non-profit organisations working in communities are needed to assist caregivers supporting children and adolescents living with HIV to achieve adherence through support groups.

It is imperative that all challenges identified be addressed and new interventions be developed and implemented. Interventions, including further research concerning these challenges, in different local settings, are required.
